# Hybrid LNP Prime Dendritic Cells for Nucleotide Delivery

**DOI:** 10.1002/advs.202303576

**Published:** 2023-10-09

**Authors:** Riddha Das, Elias A. Halabi, Ina R. Fredrich, Juhyun Oh, Hannah M. Peterson, Xinying Ge, Ella Scott, Rainer H. Kohler, Christopher S. Garris, Ralph Weissleder

**Affiliations:** ^1^ Center for Systems Biology Massachusetts General Hospital 185 Cambridge St, CPZN 5206 Boston MA 02114 USA; ^2^ Department of Pathology Massachusetts General Hospital Boston MA 02114 USA; ^3^ Department of Radiology Massachusetts General Hospital Boston MA 02114 USA; ^4^ Department of Systems Biology Harvard Medical School 200 Longwood Ave Boston MA 02115 USA

**Keywords:** cancer, dendritic cells, nanoparticles, TLR, vaccine stimulant

## Abstract

The efficient activation of professional antigen‐presenting cells—such as dendritic cells (DC)—in tumors and lymph nodes is critical for the design of next‐generation cancer vaccines and may be able to provide anti‐tumor effects by itself through immune stimulation. The challenge is to stimulate these cells without causing excessive toxicity. It is hypothesized that a multi‐pronged combinatorial approach to DC stimulation would allow dose reductions of innate immune receptor‐stimulating TLR3 agonists while enhancing drug efficacy. Here, a hybrid lipid nanoparticle (LNP) platform is developed and tested for double‐stranded RNA (polyinosinic:polycytidylic acid for TLR3 agonism) and immune modulator (L‐CANDI) delivery. This study shows that the ≈120 nm hybrid nanoparticles‐in‐nanoparticles effectively eradicate tumors by themselves and generate long‐lasting, durable anti‐tumor immunity in mouse models.

## Graphical abstract

A synthetic nanoparticle‐in‐nanoparticle strategy is developed to effectively eradicate tumors and generate long‐lasting, durable anti‐tumor immunity in mouse models. The material consisted of a hybrid lipid nanoparticle (LNP) platform for double‐stranded RNA (polyinosinic:polycytidylic acid for TLR3 agonism) and an immune modulator (cIAP inhibitor in L‐CANDI).

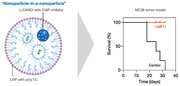

## 1 Introduction

Most cancers sustain an immunosuppressive microenvironment^[^ ^1^ ^]^ through elaborated mechanisms that are slowly being unraveled.^[^ ^2^ ^]^ Among the different immunotherapy approaches, cancer vaccination is of particular interest as it has the potential to elicit systemic and potentially durable anti‐tumor effects. Cancer therapeutic vaccines take many forms, such as peptide admixed with adjuvant, adoptive cell therapy of antigen‐loaded dendritic cells (DCs), or mRNA delivery of encoded neoantigens.^[^ ^3^ ^]^ Irrespective of the choice of tumor‐associated antigens (TAA), immunostimulatory adjuvants are needed to boost vaccine effectiveness.^[^ ^4^ ^]^ Several different adjuvants have been used, although ample room exists for improvement.

Aluminum compounds are commonly used vaccine adjuvants particularly effective at promoting humoral immune responses. Unfortunately, they are less efficacious in inducing cell‐mediated immunity, which is essential for enhanced cancer vaccine efficacy. High‐capacity cellular immunity stimulators include modified lipopolysaccharide derivatives (monophosphoryl lipid A), and double‐stranded RNA (dsRNA). Synthetic mimics of viral dsRNA include polyinoinosinic–polycytidylic acid (poly I:C), its derivative poly‐ICLC (Hiltonol, i.e., poly I:C stabilized with poly‐L‐lysine and carboxymethyl cellulose), RGC100 and ARNAX among others.^[^ ^5^ ^]^ These dsRNA mimickers are ligands of the endosomal toll‐like receptor 3 (TLR3), which is most highly expressed in cDC1, cells that are essential mediators of cell‐mediated immunity.^[^ ^6^ ^]^ The mechanism of action of these compounds has been well summarized while studies comparing their effectiveness are ongoing.^[^ ^7^ ^]^

There is considerable interest in potentiating the immune‐enhancing effects while keeping side effects to a minimum. We hypothesized that it should be feasible to enhance dsRNA‐mediated TLR3 effects through synergistic and complementary pathway activation in DC. Recent work in tumor‐associated myeloid cells has shown that dual NFkB pathway manipulation is effective and improves tumor control.^[^ ^8^ ^]^ This prior work used a 37 nm carbohydrate platform (CANDI) to deliver multiple small molecule immune modulators to tumor‐associated macrophages and a lesser degree to DCs. Here we reasoned that an analogous strategy could be developed for dendritic cell‐targeted vaccines but would have to be based on lipid nanoparticles (LNP) to improve dsRNA delivery. LNP has become the nanomaterial of choice for the delivery of nucleic acids given their considerable payload capacity, favorable pharmacokinetics, and ability to deliver cargo inside target cells. Through recent COVID vaccine efforts,^[^ ^9^ ^]^ we now have a much better understanding of the composition on nucleic acid delivery.

Here we describe the synthesis and testing of a new hybrid LNP that contains i) poly I:C and ii) an LNP internal, second nanoparticle as a “sponge” for a small molecule cIAP antagonist to enhance DC stimulation.^[^ ^8^, ^10^ ^]^ We show the synthesis of the “nanoparticle in nanoparticle” concept and that this approach effectively controls murine colorectal tumors. This novel hybrid LNP can lead to vastly enhanced efficacy over LNP/poly I:C alone.

## 2 Results

### 2.1 Synthesis of Hybrid LNP

**Figure 1:**
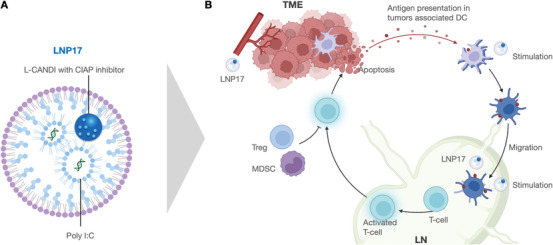
Overview. A) The hybrid LNP (left) are ≈120 nm nanoparticles that contain encapsulated poly I:C and ≈38 nm cross‐linked bi‐succinyl‐β‐cyclodextrin nanoparticles as a reservoir for cIAP inhibitor LCL‐161(CANDI‐502). Encapsulation of CANDI is facilitated by palmitoylation of the cyclodextrin nanoparticle (L‐CANDI; structures not drawn to scale). B) Hybrid LNP circulates and thus gains access to myeloid dendritic cells in the tumor microenvironment and lymph nodes. Dual stimulation via poly I:C and cIAP inhibitor leads to IL‐12 production and subsequent activation of T cells.

We conceptualized a hybrid, nanoparticle consisting of a lipid shell, internal poly I:C as a TLR3 immunostimulant, and a smaller, cross‐linked cyclodextrin nanoparticle (CANDI) with a therapeutic payload of a cIAP inhibitor (**Figure** **1**). The choice of the active ingredients (TLR3 agonist and cIAP inhibitors) was a deliberate one. The TLR3 agonist Poly I:C was chosen as an immune adjuvant given its clinical use.^[^ ^11^ ^]^ The cellular inhibitor of apoptosis protein 1 (cIAP) inhibitors had previously been shown to activate myeloid cells via the non‐canonical NFkB pathway.^[^ ^10^, ^12^ ^]^

This design of a “nanoparticle in a nanoparticle” (NIN) has several advantages over simply loading small molecule drugs into LNP based on their partition coefficient. For one, the NIN concept allows tight integration of small molecules with better controllable release kinetics over direct encapsulation. This is particularly advantageous for small molecules with a narrow therapeutic window or considerable off‐target toxicity, such as free small molecule cellular inhibitor of apoptosis protein 1(cIAP) compounds used here. While it is theoretically possible to load small amounts of LCL‐161 into LNP, we found that LCL‐161 is rapidly released (Figure S3, Supporting Information). The construction of a “nanoparticle in a nanoparticle” thus had considerable advantages.

To internalize the CANDI as a reservoir for the model cIAP inhibitor LCL‐161, we first modified the CANDI surface with palmitic acid to yield L‐CANDI. Prior research has shown that CANDI with LCL‐161 activates the non‐canonical NFkB pathway that stimulates DC.^[^ ^10^ ^]^ The LNP was designed to have a long circulation half‐life so that the nanoparticle would accumulate in phagocytic myeloid cells in the tumor microenvironment and its draining lymph nodes. Once there, the LNP would be phagocytosed, leading to an upregulation of immunostimulatory molecules, including the cytokine IL‐12. This, in turn, would activate lymphoid cells resulting in theoretical anti‐tumor functions. The current research was designed to optimize LNP synthesis, characterize different preparations, and then perform in vivo efficacy and mechanistic studies in mouse models of cancer.

The LNPs were designed using well‐established components and molar ratios resembling those of published LNP, including C12‐200 lipid^[^ ^13^ ^]^ since their biodistribution and pharmacological properties are well understood. We hypothesized that these LNPs also show affinity to phagocytic cells, including dendritic cells, and could thus be used for immune stimulation. Prior studies had explored the surface functionalization of LNP with DC targeting ligands, antibodies or PEG moieties,^[^ ^14^, ^15^, ^16^, ^17^ ^]^ contributing to synthetic complexities. Moreover, such altered surfaces can negatively affect biological properties.^[^ ^18^ ^]^ In contradistinction, our strategy offers a modular system to activate DCs, circumventing the need to functionalize LNPs and providing a simple and scalable strategy for DC‐based cancer therapy.

Initial feasibility studies were carried out to optimize the encapsulation of poly I:C and L‐CANDI carrying LCL‐161. The final preparation (LNP1) consisted of C12‐200 (Figure S2, Supporting Information), DSPC, cholesterol, and DMG‐PEG2000 at a molar composition of 35:16:46.5:2.5. Table S1 (Supporting Information) provides an overview of the different LNP formulations synthesized. LNPs 2–13 primarily represent experimental versions in which polyglutamic acid (PGA) was initially used as an inexpensive charge condenser to explore the biological properties of different formulations. LNPs 14–17 were subsequently designed to contain poly I:C and it is those preparations that form the basis of this report. LNP14 contained a 1:10 w/w ratio of poly I:C and C12‐200, and resulted in a stable LNP formulation (Figure S5, Supporting Information). We next proceeded to co‐loading this preparation with L‐CANDI that contained LCL‐161 resulting in the “nanoparticle‐in‐a nanoparticle” preparation termed LNP17.

We also synthesized LNP17 versions for imaging. For fluorescence imaging (LNP17Fl) we used Alexa fluor‐555 stained L‐CANDI and/or Atto647N stained LNP (details are in Experimental Section). For electron microscopy (EM) we used 2% uranyl acetate stained L‐CANDI (LNP17EM). LNPs demonstrated encapsulation efficiency of 75% ± 5% for poly I:C, as determined using the Ribogreen assay, and 18.6% ± 2.2% for L‐CANDI, as demonstrated using inclusion complexation assay (Figure S4, Supporting Information). We determined 10:1 w/w L‐CANDI: C12‐200 as the optimal loading ratio as indicated by complete encapsulation. Overall, the optimized LNP17 contained 16% lipid, 1% poly I:C, 82% L‐CANDI, and 1% LCL‐161 by weight. The overall synthetic yield was 70%.

### 2.2 Characterization of Hybrid LNP

**Figure 2:**
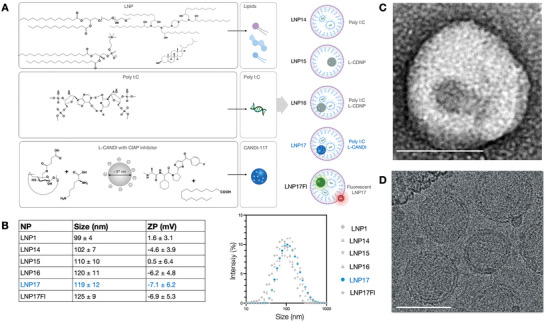
Composition of different hybrid LNP. Overview of different LNP preparations synthesized. A) LNP17 represents the fully functional hybrid “nanoparticle‐in‐nanoparticle.” LNP17Fl contains additional fluorochromes to investigate pharmacokinetics by intravital imaging but is otherwise similar to LNP17. B) On average, LNPs are approximately 120 nm in mean diameter and are negatively charged. C) Transmission electron microscopy images of LNP17EM (≈120m nm) with a single uranyl acetate stained L‐CANDI nanoparticle incorporated (≈38 nm, scale bar = 100 nm). D) Cryo‐EM without stains shows the double‐layer LNP membrane. L‐CANDI is not visible with this method. Scale bar = 100 nm.

**Figure** **2** and Table S1 (Supporting Information)provides an overview of the different LNP formulations synthesized. In short, LNP14 only contained poly I:C (0.1 mg mL^−1^), LNP15 only contained L‐CANDI (25 mg mL^−1^), LNP16 contained poly I:C (0.1 mg mL^−1^) and L‐CANDI (25 mg mL^−1^), and LNP17 contained poly I:C^−^ and LCL‐161 (0.1 mg mL^−1^) loaded L‐CANDI. LNP17Fl is a fluorescent version of LNP17. The different LNP preparations had a mean particle size between 100 and 125 nm diameter. The zeta potential was negative for all preparations tested (range −4 to −7 mW). Preparations were stored at 4 °C and used for experiments within 3 days of synthesis. Electron microscopy (EM) was performed to elucidate the structure of the hybrid LNP. Cryogenic electron microscopy (Cryo‐EM) of hybrid LNPs (LNP17) showed a typical double‐layer membrane similar to what has been described for other LNP formulations.^[^ ^19^, ^20^, ^21^ ^]^ Since CryoEM did not reveal internal structures due to low electron density, we used regular TEM with uranyl acetate‐stained L‐CANDI (LNP17‐EM). This confirmed the presence of L‐CANDI inside the hybrid LNP (Figure 2).

### 2.3 Screening for Efficacy

**Figure 3:**
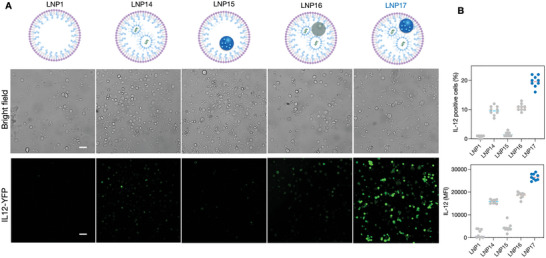
IL‐12 induction in dendritic cells. A) Myeloid cells were isolated from the bone marrow of IL‐12‐eYFP mice. Myeloid precursor cells were differentiated into dendritic cells (DC) using Flt3L and then used for screens with different formulations. Aliquots of DC received LNP preparations as indicated (LNP1: 2 µg lipids; LNP14: 2 µg lipids, 0.1 µg poly I:C; LNP15: 2 µg lipids, 0.1 µg LCL‐161; LNP16: 2 µg lipids, 0.1 µg poly I:C; LNP17: 0.1 µg poly I:C /0.1 µg LCL‐161 per 105 cells; for other dosages, see Figure S6, Supporting Information). Scale bar = 50 µm. B. Note that IL‐12 induction is the highest in the dually loaded LNP17 preparation (*p* < 0.001 for DC) and much higher than either mono‐preparation alone (N = 9 replicates). LNP17 leads to high IL‐12 induction in roughly 20% of cells at the current concentration tested.

Having created a series of different nano‐formulations, we next set out to screen for their capacity of IL‐12 induction in bone marrow‐derived cells from donor mice. A number of different quality control measures were implemented to assure LNP stability (see Experimental Section). We used IL‐12‐eYFP donor mice for these experiments and isolated bone marrow cells through femoral flushes. Isolated cells were then cultured and differentiated into DCs by treatment with Flt3L over 10 days. Cells were incubated with different amounts of LNP and IL‐12 induction was measured by eYFP fluorescence. **Figure** **3** shows that LNP17 had the highest IL‐12 induction (determined by median fluorescent intensity (MFI) or as % of IL‐12 positive cells). While these experiments were performed with similar molar amounts of reagents to compare different preparations, we also performed dose‐response and toxicity experiments to determine therapeutic windows. Figure S6 (Supporting Information) demonstrates a dose‐response study, eliciting detectable IL‐12 production in ≈20% of all DC at sub‐toxic doses (Figure 3).

### 2.4 Pharmacokinetics (PK) and In Vivo Efficacy

**Figure 4:**
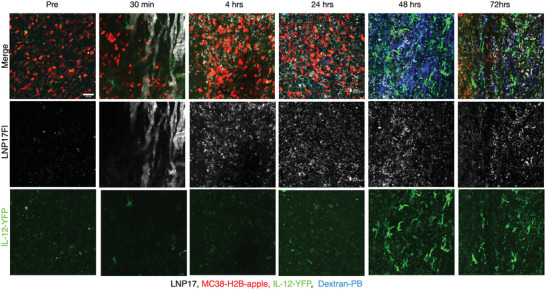
Intravital imaging to determine pharmacokinetics and dynamics. Dorsal window chambers implanted into B6 mice were inoculated with MC38‐H2Bapple tumor cells. Seven to 10 days later, LNP17Fl (fluorescent version of LNP‐17) was injected to determine intratumoral distribution. The LNP is initially seen in tumor microvasculature but accumulates in non‐tumor cells at 4 h. At 24 h, all LNP17Fl is associated with cells and can be seen as puncta at this low resolution (See Figure S9, Supporting Information, for higher resolution). Note the marked IL‐12 induction at 48 and 72 h, mostly in cells with dendritic morphology and motility. Furthermore, a significant reduction in red tumor cells at later times indicates a reduction in tumor cell abundance. Scale bar = 100 µm.

Having identified the optimum preparation and dose for IL‐12 induction in dendritic cells, we next set out to perform in vivo experiments to test the anti‐tumor efficacy of LNP17. LNP17 had a vascular half‐life of roughly 30 min (Figure S7, Supporting Information) and accumulated primarily in the liver, spleen, tumor, and lymph nodes 24 h after administration (Figure S8, Supporting Information). To determine the kinetics of cellular accumulation within the tumor microenvironment, we performed intravital microscopy experiments using the MC38 tumor dorsal window chamber model (**Figure** **4**). These experiments allowed single‐cell resolution PK/pharmacodynamics (PD) measurement and showed that the materials primarily accumulated in tumor‐associated DCs and macrophages. As expected, IL‐12 induction in the cells was time‐dependent, with a maximum effect of ≈72 h after IV administration. IL‐12 signals were the brightest in cells with dendritic morphology, consistent with our prior observations. Interestingly, longer observation of LNP17‐treated tumors showed substantial decreases in tumor cells in imaging experiments that coincided with the induction of IL‐12 in the tumor microenvironment.

**Figure 5:**
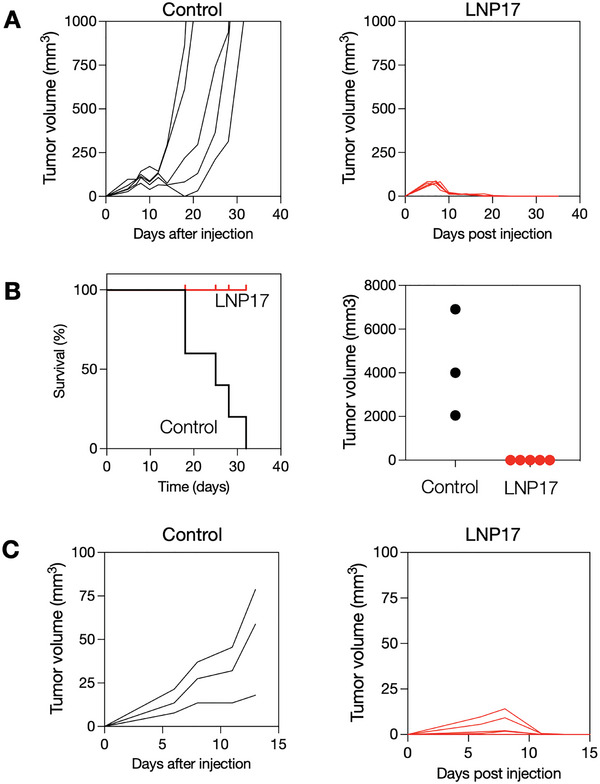
In vivo efficacy in MC38 tumors. BL6 mice received MC38 tumors on day 0 and were allowed to grow to ≈100 mm^3^ established tumors. By day 7, mice received a single IV injection of LNP17 (N = 5; 50 µL LNP containing 5 µg poly I:C and 5 µg LCL‐161/mouse) or PBS as control (N = 5). Mice were sacrificed on day 30 or when tumors had grown >1500 mm^3^. A) Comparison of tumor growth in LNP17 versus PBS control‐treated mice. B) Survival curve of different mouse cohorts. Right: tumor volumes on day 19. Note the remarkable efficacy in rejecting tumors. C) The treated mice (N = 5) were re‐challenged s.c. with MC38 cancer cells two months after primary treatment to test for memory responses and control mice (N = 3) were compared. Note the tumor rejection in previously treated mice indicating memory T cell response.

Having determined the biodistribution and cellular accumulation, we next set out to determine therapeutic efficacy. For these experiments, we implanted MC38 tumors into the flank of BL6 mice. One week after implantation, mice received a single intravenous injection of LNP17 (containing 5 µg poly I:C and 5 µg LCL‐161 per mouse). At the injection time, tumor volume in both groups was ≈100 mm^3^. Tumor volumes were subsequently measured by calipers, and survival was recorded. **Figure** **5** summarizes the in vivo results. This data shows complete tumor control in LNP17‐treated animals. Within 1 and 2 days, we observed a ≈30% and 80% reduction in tumor volume, respectively, similar to the results we observed by intravital imaging (Figure 4). By day 18, tumors in LNP17‐treated mice were completely eradicated while control tumors rapidly progressed and reached a volume of ≈1000 mm^3^. These results led to a 100% long‐term survival of LNP17‐treated animals, with all control‐treated animals dead 32 days after tumor implantation. To test long‐term anti‐tumor memory responses, we took complete responders from our earlier LNP17 treatment cohort and re‐challenged them subcutaneously on contralateral flanks with MC38 tumor cells 2 months after they had rejected their primary tumors. Naive BL6 mice that had never seen MC38 nor LNP17 were used as controls. Naive mice had progressive tumor growth. However, re‐challenged mice controlled their tumor growth and had complete eradication of their secondary tumors within 2 weeks post re‐challenge, demonstrating a memory response.

**Figure 6:**
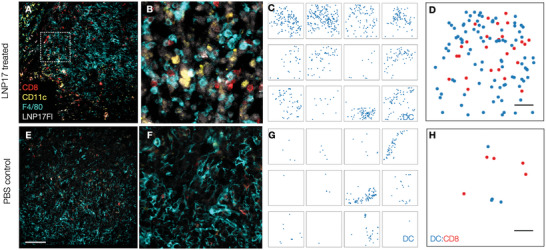
Dynamic changes in the tumor immune microenvironment induced by LNP17. A–D) Representative images of MC38 tumor with LNP17 treatment or E–H) PBS control. A,E) Low‐resolution cyclic imaging of TME with stains for CD8 (red), CD11c (yellow), F4/80 (cyan), and LNP17Fl (white) are shown. Scale bar = 100 µm. B,F) The middle images are higher resolution subsets to show the dense CD11c and CD8 T cell infiltration. C,G) Image analysis of 12 different fields of views of C) treated or G) untreated tumors. Each DC is shown as a blue dot to demonstrate the heterogeneity across different FOV. Note the higher DC infiltration in LNP17‐treated tumors. D,H) Spatial call localization of DC (blue) and CD8 (red) in D) treated and H) untreated tumors. Note the much higher frequency of DC and CD8 interactions and close proximity in LNP17Fl‐treated tumors. Most of the CD8 in untreated tumors are exhausted. Scale bar = 100 µm. For additional information see Figures S10 and S11 (Supporting Information).

To pinpoint to potential mechanism of action, we performed a histological analysis of tumors (**Figure** **6**). We examined immune populations in either LNP17‐treated or untreated tumors and observed a substantial increase in tumor DCs (CD11c+ cells) and CD8 T cells (CD8+ cells) after LNP17 therapy. Proportions of tumor‐associated macrophages (F4/80+ cells) and overall immune cell numbers (CD45+) were essentially unchanged. Of note, we observed co‐clustering of DCs and CD8 T cells in LNP17‐treated tumors; which was not the case in untreated tumors.

## 3 Discussion

The current study shows that high‐efficiency hybrid LNP (“nanoparticle‐in‐nanoparticle”) can be synthesized to dually stimulate DCs via different cellular pathways and that this stimulation can be sufficient by itself to provide tumor control. We used a commonly employed LNP formulation to encapsulate i) low molecular weight poly I:C for stimulation of the TLR3 pathway and ii) lipophilic cyclodextrin nanoparticles as a reservoir for small molecule payloads to affect complementary, immune stimulatory pathways inside DC. In particular, we started with CANDI^[^ ^8^ ^]^ to entrap a cIAP inhibitor (LCL‐161) since this class of agents had previously been shown to activate myeloid cells via the non‐canonical NFkB pathway.^[^ ^10^, ^12^ ^]^ To efficiently internalize the ≈38 nm CANDI nanoparticle into LNP, we modified the cyclodextrin nanoparticle surface with different lipids and found palmitic acid to be the most efficient. We estimated that a mean LNP (120 nm) thus had ≈1 CANDI nanoparticle (Figure 2C), each with an estimated maximum capacity of ≈1500 molecules of LCL‐161 (Figure S4, Supporting Information) and ≈100 molecules of poly I:C calculated by using a molecular volume model according to previously published literature.^[^ ^22^ ^]^ Fluorescent versions were used to determine the PK and cellular distribution. These data show that LNP17 had a T_1/2_ of 30 min (Figure S7, Supporting Information) and accumulated in the tumor, lymph nodes, and liver (Figure S8, Supporting Information). These favorable PK likely contributed to the overall efficacy of the approach.

Our approach differs from prior approaches. Poly I:C has long been used as a stand‐alone therapeutic agent,^[^ ^23^ ^]^ unfortunately with considerable side effects,^[^ ^24^ ^]^ the use of poly I:C as an immune adjuvant is more recent. Most trials use the material as an adjuvant in peptide or DC vaccination programs. Some trials have evaluated a stable form of poly I:C stabilized with poly‐L‐lysine and carboxymethylcellulose, Hiltonol.^[^ ^25^, ^26^ ^]^ Additional trials have tested combinations of poly‐ICLC with other immune stimulators such as granulocyte‐macrophage colony‐stimulating factor, resiquimod, or Montanide‐ISA‐51.^[^ ^11^, ^27^, ^28^, ^29^ ^]^ These trials found that poly I:C and poly‐ICLC effectively contribute to host anti‐tumor responses as immunostimulatory components of cancer vaccines. However, many of these vaccination approaches relied upon an admixture of antigen and adjuvant. Prior direct immunotherapy studies in mouse models of melanoma used doses of poly I:C upward of 200 µg to see anti‐tumor therapeutic effects.^[^ ^30^ ^]^ In contrast, our LNP‐based delivery approach achieves complete tumor eradication using doses of 5 µg with single administration, demonstrating a 40× increase in potency with our nanoformulation. This is important as currently used vaccination protocols in clinical trials are limited in their application of efficient nanoformulations to potentiate vaccine efficacy. For example, NeoVax trials in melanoma using patient‐specific neoantigen peptides now rely upon the admixture of synthetic peptides with poly‐ICLC.^[^ ^31^ ^]^ While these trials still demonstrated therapeutic responses, they suggest substantial room for improvement. More recent approaches have used intravenously administered mRNA encoded personalized neoantigens in LNP vectors, which showed remarkable responses in difficult‐to‐treat pancreatic cancer.^[^ ^3^ ^]^

In the current study, we show the efficacy and lack of toxicity of the hybrid LNP immunostimulant at pharmacological concentrations. LNP themselves may be near optimal vectors to stimulate cell‐mediated immunity inducing DCs (cDC1) as cDC1 homeostatically mature in response to apoptotic cells and normally engender immune tolerance.^[^ ^32^ ^]^ LNP themselves can trigger DC immunoregulatory maturation through cholesterol pathways, though poly I:C loaded LNP can trigger immunogenic programs in DC.^[^ ^32^ ^]^ Several future experiments would be logical extensions to further improve effectiveness. For example, it is possible to further load the CANDI nanoparticle with additional small molecule payloads to induce DC3, and type 1 cell‐mediated immunity, effector pathways (e.g., IL‐12, CXCL9/10, IFNG). Prior research had shown that dual stimulation in macrophages via the canonical NFkB (e.g., TLR7/8 agonists such as R848) and non‐canonical NFkB pathway (e.g., cIAP inhibitors) could achieve this.^[^ ^8^ ^]^ There is further evidence that STAT, PI3Kg, or JAK inhibition could re‐program tumor myeloid cells and potentiate the effect. Second, it is possible to target the hybrid LNP to DC through the use of antibodies (e.g., CD8A, CLEC9A, ITGAE, ITGAX, CD141, or XCR1). Such antibodies could be anchored on the surface of hybrid LNPs. Third, it is possible to arm LNP with tumor‐associated antigens, including peptides or mRNA expressing tumor antigens. Irrespective of these additional modifications, the current first generation of hybrid LNP is already a highly efficient dendritic cell stimulant.

## 4 Experimental Section

### Materials

The solvents and reagents were purchased from Sigma–Aldrich and Thermo Fisher. Citrate buffer was purchased from Boston BioProducts, Inc. All the lipids were purchased from Avanti Polar Lipids. MilliQ water obtained from Waters filtration system was used for all experiments. Pharmacological payloads poly I:C and LCL‐161 were purchased from InvivoGen and MedChem Express, respectively. Antibodies were purchased from BioLegend, BD Biosciences, and Thermo Fisher.

### Synthesis of LNPs

The LNPs were prepared by rapidly mixing an ethanol solution of lipids and an aqueous solution of therapeutic payloads with a microfluidic device, followed by dilution with PBS. The lipid solution comprised of C12‐200, cholesterol (Sigma–Aldrich), DSPC (Avanti Polar Lipids), and mPEG2000‐DMG (MW 2500, Avanti Polar Lipids) at a molar ratio of 35:16:46.5:2.5 in ethanol (by volume). For fluorescent LNPs (LNP17Fl), 0.5 mol.% of ATTO647N‐DOPE was added to the ethanol solution resulting in the final formulation of 35:15.5:46.5:2.5. For all LNPs, the aqueous solution was mixed in a 3:1 water:ethanol volume ratio. For LNPs without any therapeutic payload (LNP1 and LNP17Fl), 10 mm sodium citrate buffer of pH 6 (Boston BioProducts, Inc) was used as an aqueous solution. For LNP14, the aqueous solution was prepared by diluting poly I:C (0.2‐1 kb, InvivoGen) in 0.5 mm sodium citrate buffer (Boston BioProducts, Inc) resulting in a final concentration of 0.1 mg mL^−1^ in LNPs. To prepare LNPs, the lipid solution and aqueous solution were injected simultaneously into the microfluidic device^[^ ^33^ ^]^ using a two syringe pumps (Aladdin pump series, AL‐4000) at a volumetric flow rate of 1:3. Ethanol and non encapsulated payloads were removed by dialyzing LNPs with PBS using 100 kDa MWCO filter membranes (Spectrum Laboratories, Inc.), then concentrating the LNPs using centrifuge filters (Amicon, 100 kDa MWCO filters, 2000 rpm). LNPs generated were ≈120 nm in size.

### Synthesis of Lipophilic Cyclodextrin Nanoparticles (L‐CANDI)

Cyclodextrin nanoparticles (CANDI) were synthesized per the previously reported method.^[^ ^8^ ^]^ Briefly, succinyl‐β‐cyclodextrin was dissolved in MES buffer (6 mL, 50 mm, pH = 5) and activated with *N*‐(3‐(dimethylamino)propyl)‐N′‐ethyl carbodiimide hydrochloride (EDC) (Fisher; 1.5 g, 10.0 eq. to carboxylate) and *N*‐hydroxysuccinimide NHS (Sigma; 550 mg, 5.0 eq. to carboxylate) for 30 min at 25 °C. A solution containing *L*‐lysine (Sigma; 70 mg, 0.5 eq to carboxylate) in MES buffer (1.5 mL) was added for cross‐linking. Ethanol purified precipitate was filtered through a 0.22 µm centrifugal filter (VWR) and purified with 10 kDa MWCO centrifugal filters (Amicon; 10,0000 g for 8 min).

To render the surface of the nanoparticles hydrophobic (L‐CANDI), 3 mg palmitic acid N‐hydroxysuccinimide ester was added to 50 mg CANDI followed by shaking for 90 min at 37 ^°^C. The reaction product was filtered using Amino centrifugation filters (10 kDa MWCO filters, 10,000 rpm). LCL‐161 (0.5 mg, payload) was dissolved in DMSO (10 µL) and subsequently added to the L‐CANDI (5 mg) to achieve a final concentration of 25 mg mL^−1^ (5% DMSO). The solution was vortexed and mixed until the solution became fully translucent. Excess LCL‐161 was filtered off using a 0.22 µm spin‐X centrifuge tube filter (VWR). Particle size and surface charge of L‐CANDI were determined by dynamic light scattering and zeta potential, respectively (Malvern, Zetasizer APS), and were prepared fresh before encapsulating in LNPs. The size of L‐CANDI was 38.2 ± 0.1 nm with zeta potential of −5 mV (Figure S1, Supporting Information).

### Synthesis of C12‐200

In a 15 mL scintillation vial equipped with a magnetic stirrer 1,2‐epoxydodecane (2 g, 11 mmol, 7 eq, Sigma) and N1‐(2‐(4‐(2‐aminoethyl)piperazin‐1‐yl)ethyl)ethane‐1,2‐diamine (330 mg, 1.57 mmol, 1 eq, Ambeed) were loaded, sealed and heated to 80 **^°^** C under constant stirring.^[^ ^34^ ^]^ The reaction was sequentially monitored by liquid‐chromatography mass‐spectrometry (LC‐MS) using an ELSD source to follow the formation of the desired product with an average mass corresponding to the mass‐to‐charge ratio (m/z) of M^+^ = 1137, and retention time = 3.63 min, ≈48 h. The crude mixture was purified by normal phase chromatography on silica gel with a solvent gradient (CH_2_Cl_2_ → CH_2_Cl_2_/CH_3_OH/NH_4_OH, 87.5:11.0:1.5, and 20 min) to yield the lipidoid C12‐200 as a pale yellow viscous oil (990 mg, 55%). ^1^H NMR (400 MHz, CDCl_3_): δ 5.00–4.00 (br s, 5H), 3.62 and 3.55 (br s, 5H), 3.00–2.00 (m, 30H), 1.52–1.25 (m, 90H), 0.87 (t, 7.2 Hz, 15H).

### Synthesis of Hybrid Nanoparticle‐in‐Nanoparticle (LNP17)

The lipid solution was similar to the LNP1 formulation mentioned above, except that L‐CANDI was incorporated in the aqueous solution to prepare the hybrid LNPs (LNP15–17). For LNP15, LNP16 L‐CANDI (25 mg mL^−1^) was diluted in 0.5 mm sodium citrate buffer. For LNP17, the aqueous solution was prepared by diluting poly I:C and L‐CANDI with LCL‐161 in 0.5 mm sodium citrate buffer resulting in a final concentration of 0.1 mg mL^−1^ poly I:C and LCL‐161 in LNPs. For LNP16, poly I:C was incorporated in the aqueous solution to the final concentration of poly I:C to resemble the negative charge of hybrid LNPs. Similar to the LNP synthesis protocol mentioned above, the hybrid LNPs were dialyzed in PBS using 100 kDa MWCO filter membranes, followed by concentrating the LNPs using centrifuge filters. The hybrid LNPs generated through the microfluidic device controlled by two syringe pumps (Aladdin pump series, AL‐4000) were ≈120 nm in size.

### Measurement of Encapsulation Efficiency of Poly I:C in LNP

Encapsulation efficiency (EE%) was measured using a fluorescence plate‐based assay using the Ribogreen reagent (Thermo Fischer). Briefly, LNPs were diluted in TE (Tris EDTA) and TE/TX (1% triton‐X in TE) buffer and incubated for 30 min at 300 rpm at 37 °C. Simultaneously, standard curves for poly I:C in TE and TE/TX buffer were generated. Subsequently, Ribogreen was added to the samples and the standard dilutions, incubated for 10 min, followed by fluorescence intensity measurement (λex: 485 nm, λem: 515 nm). Using the standard curves, the poly I:C concentration was quantified in TE and TE/TX buffer. EE% was calculated as the difference between the total RNA and non‐encapsulated RNA divided by the total RNA.

### Quantification L‐CANDI into LNPs

The amount of L‐CANDI incorporated into the LNPs was quantified with a fluorescence assay using bisbenzimide H 33258 (Hoechst staining solution, Sigma, 0.5 µg mL^−1^) as an indicator of inclusion‐complex formation selective only to the CD units in L‐CANDI. Figure S4A (Supporting Information) illustrates the proof‐of‐concept experiment and the results. Briefly, in the presence of 𝛃‐cyclodextrin units, the Hoechst molecules interlocked to induce an increase in fluorescence emission (reminiscent of DNA mechanism). The fluorescence emission (λex = 348 nm, λem = 510 nm) of solutions containing a fixed amount of Hoechst (0.05 ng mL^−1^) and a variable amount of L‐CANDI (100–3200 µg) was used to correlate the fluorescence intensity to amount of L‐CANDI (µg) Figure S4B (Supporting Information). Solutions containing LNP1 (no L‐CANDI) and LNP15 (with L‐CANDI, no poly I:C) were analyzed at a fixed concentration (1 mg mL^−1^, 100 µL). The difference between the detected fluorescence emission for LNP15 and LNP1 after background subtraction from the PBS control, corresponded to the amount of L‐CANDI in the LNP15 solution (≈930 µg). We estimated 18.6% ± 2.2%encapsulation efficiency (EE) from the ratio of final L‐CANDI concentration with the initial amount of L‐CANDI used to construct LNP15 (5000 µg, 200 µL, 25 mg mL^−1^) Figure S4C (Supporting Information). All experiments were done in triplicates (N = 3) and compared using an unpaired student's t‐test.

### Characterization of LNP

Particle size and surface charge for the LNPs were determined by dynamic light scattering and zeta potential, respectively (Malvern, Zetasizer APS). The stability of the LNPs was determined in serum. The size and surface charge was measured after incubating LNPs in media with 10–50% serum at 37 °C for 4 h. Fluorescently labeled LNPs were further characterized using a fluorescence microplate reader (Tecan) with fluorescence intensity measured at excitation and emission wavelength of 646 and 664 nm respectively.

### Quality Control of LNP

A number of quality control studies of the LNP formulations were performed prior to in vitro and in vivo use. Wherever possible, LNP preparations were prepared fresh and used them within 3 days to synthesis. For longer‐term analysis, serial stability testing was performed (Figure S5, Supporting Information) by determining size, zeta potential, and poly‐dispersity index in PBS and in the presence of serum at 4 °C. Endotoxin contamination was determined by the limulus amoebocyte lysate (LAL) test which did not show any detectable endotoxin in LNP‐17. Additional serial experiments included periodic flow cytometry with appropriate controls and microscopy.

### Electron Microscopy

Cryo‐EM and TEM were used to characterize the hybrid LNP. For cryo‐EM, 5 µL of a fresh LNP sample was added to glow‐discharged Quantifoil holey carbon grids (Cu R1.2/1.3; 400 mesh) in a Vitrobot chamber (Vitrobot markIV, Thermo Fisher Scientific) at ≈100% relative humidity and allowed to rest for 30 s. The grid was then blotted with Whatman filter paper for 3 s, and then the sample was plunged into liquid ethane cooled by liquid nitrogen. The frozen grids were then assembled into cassettes and stored in liquid nitrogen until imaging. Cryo‐EM imaging was done using ThermoFisher Scientific Talos Arctica operating at 200 kV. For TEM imaging, 5 µL of the concentrated hybrid LNPs were added to glow discharged carbon coated 400 mesh Cu grid. Excess liquid was adsorbed using a Whatman filter paper. The sample was stained with 2% uranyl acetate (Electron Microscope Sciences) for 1 min. For TEM imaging of LNP17, L‐CANDI (25 mg mL^−1^) was stained with 2% uranyl acetate solution at 37 °C for 5 min under constant stirring at 600 rpm (Thermomixer, Eppendorf). The stained solution was washed with H_2_O (3×, 300 µL) using a 10 kDa MWCO filter membrane (5 min, 10,000 rpm). The stained L‐CANDI was diluted to ≈25 mg mL^−1^ with H_2_O and used to construct the stained LNP17. Imaging was done using Phillips CM10 operating at 100 kV.

### Bone Marrow‐Derived DC Screens

Bone marrow was isolated from the femurs and tibias of a freshly euthanized IL‐12‐eYFP mouse. Briefly, bones were cut open at both ends in a laminar flow hood, and cells were flushed out into a centrifuge tube using sterile PBS. Red blood cells (RBCs) were lysed using RBC Lysis buffer (BioLegend). Subsequently, cells were counted using an automatic cell counter (Invitrogen Countess 3) and seeded in a 96‐well iBIDI plate (100k/well) for imaging and 24 well plates (250k/well) for FACS analysis in the presence of recombinant murine GM‐CSF (Biolegend) and recombinant human Flt3L‐Fc (BioLegend). For macrophages, cultures were differentiated for 5 days, with GM‐CSF media changed every 2 days. Cultures were differentiated for 9 days for DC, with Flt3L‐FC and GM‐CSF media changed after 5 days. Cells were treated with different dosing of LNPs ranging from 0.5 ng to 0.1 µg of poly I:C/well in triplicate. Imaging for IL‐12‐eYFP signals was performed after 24 h using an automated Olympus screening microscope (BX63) and analyzed using Attune NxT flow cytometer (Thermo Fisher).

### Flow Cytometry

The BMDCs were collected into polystyrene round bottom Falcon tubes (Thermo Fisher) and were centrifuged to remove the supernatant fluid at 300 rcf for 5 min. Cells were then resuspended in FACS buffer (PBS with 2 mm EDTA and 2% Fetal Calf Serum) and stained with Fc block (Biolegend) and fluorochrome‐conjugated antibodies (Table S2, Supporting Information). The organs were harvested and mechanically dissociated for biodistribution studies using surgical scissors. Collagenase IV at 0.2 mg mL^−1^ in RPMI 1640 was added to the tissues, followed by vigorous shaking at 37 °C for 45 min. After digestion, tissues were filtered through a 40 µm cell strainer and resuspended in protein‐free PBS. Cells were stained using AquaAmine Live Dead Fixable viability stain (Thermo Fisher) and fluorochrome‐conjugated antibodies (Table S2, Supporting Information). The cells/tissues were washed using FACS buffer by centrifugation and were resuspended in 300 µL FACS buffer at 4 °C until the analysis. Sample data were acquired using an Attune NxT flow cytometer (Thermo Fisher), and data were analyzed using FlowJo 10 software (TreeStar).

### Toxicity Assay

Immortalized macrophages (iMac) were seeded at 10000 cells per well in 96‐well plates 24 h before the experiment. Cells were washed with PBS buffer and then treated with different dosings of LNPs ranging from 0.5 ng to 1 µg of poly I:C/well in triplicate. After 24 h of incubation, the cells were then wholly washed off with PBS buffer three times, and 10% Alamar blue in serum‐containing media was added to each well (220 µl) and further incubated at 37 °C for 2 h. Cell viability was then determined by measuring the fluorescence intensity at 570 nm using a fluorescence microplate reader (TECAN).

### Animal Studies

Various mouse models were used to study different aspects of LNP delivery and therapeutic efficacy. The MGH Institutional Animal Care and Use Committee (IACUC) approved all studies (animal protocol no. 2021N000272). A total of N = 104 mice were used. This included immunocompetent C57BL/6 mice (Jackson Laboratories) for biodistribution (N = 5) and tumor implantations (N = 20). IL‐12‐eYFP mice were used for IL‐12 experiments, including bone marrow harvesting and intravital imaging. We used MC38‐WT cells (2,000,000 cells/mice) implanted subcutaneously into C57BL/6 mice (N = 10 for each tumor model) for tumor implantations, using a 10 µL Hamilton micro‐syringe.  The tumor size was monitored every 24 h using slide calipers. When the tumors had grown to around 100 mm^3^, mice received an IV administration hybrid LNP. The dose was 50 µL LNP containing 5 µg poly I:C and 5 µg LCL‐161. All doses were given via the tail vein. The tumors were measured every 48 h using slide calipers until the tumors in control mice had grown to 500 mm^3.^ The LNP‐treated mice (N = 5) were re‐challenged with MC38‐WT 60 days after the first implantation. For the re‐challenge experiment, MC38‐WT cells (2,000,000 cells/mice) were implanted subcutaneously into previously LNP17‐treated mice or control C57BL/6 mice (N = 3).

### Intravital Imaging

Mice (male/female, 8 weeks) were anesthetized for window chamber implantation and subsequent imaging using ketamine/xylazine and 1% isoflurane. IL‐12‐eYFP C57Bl/6J mice were implanted with a dorsal skin fold chamber and MC38 cells expressing H2B‐apple (1000000 cells) were injected into the dorsal skin fold chamber. Tumor growth was monitored by confocal imaging. At 10 to 14 days after implantation, 60 µl of fluorescent LNP was injected via tail vein. Before and after treatment with LNP, images were collected using identical image settings as the IL‐12‐eYFP imaging. Changes in IL‐12‐eYFP and LNP fluorescence distribution and intensity were monitored for up to three days.

Confocal images were collected using a customized Olympus FV1000 confocal microscope (Olympus America) with a XLUMPlanFL N 20x (NA 1.0) water immersion objective (Olympus America). IL‐12‐eYFP, MC38‐H2B‐apple tumor cells, LNP, and a vascular probe (Dextran‐pacific‐blue) were excited sequentially using a 405 , a 473, a 559, and a 633 nm diode laser, respectively, in combination with a DM‐405/488/559/635 nm dichroic beam splitter (Olympus America). Emitted light was further separated by beam splitters (SDM‐473, SDM‐560, and SDM‐640) and emission filters BA430‐455, BA490‐540, BA575‐620, and BA655‐755 (Olympus America). Confocal laser power settings were carefully optimized to avoid photobleaching, phototoxicity, and damage to the tissue. All images were processed using Fiji (ImageJ2, Vers.2.3/1.54d).

### Multiplexed Immunofluorescence

MC38 tumors were allowed to grow for 7–10 days to reach substantial size for LNP17 treatment and harvested on the 3rd day after the intravenous injection of fluorescent LNP17. Control mice were injected with PBS. Harvested tumors were fixed in 4% PFA overnight and incubated overnight in 30% sucrose before embedding in OCT to generate frozen sections. For multiplexed immunofluorescence imaging, frozen cells were dried at room temperature for 10 min and blocked with Intercept blocking buffer (Li‐Cor Biosciences) for 30 min. Antibodies against target proteins were conjugated to custom‐designed imaging probes for cyclic imaging to enable a multiplexed analysis of target proteins from each tissue section as previously described.^[^ ^8^, ^35^, ^36^ ^]^ The antibodies used for imaging are summarized in Table S3 (Supporting Information). For each sample, 15–26 fields of view were collected to image most of the tumor area. An Olympus BX‐63 upright automated epifluorescence microscope was used to acquire fluorescent images. DAPI, FITC, Cy3, and Cy5 filter cubes were used to excite DAPI, AF488, AF555, and AF647, respectively.

### Image Analysis

Images taken throughout image cycles were registered by the virtual stack slices registration plugin of FIJI. The DAPI staining of the last cycle was used for cell segmentation by Cell Profiler (Ver 4.1.3). Quenched images were subtracted from the images of subsequent cycles for background subtraction before the quantification of the fluorescent intensity of each protein marker. The positivity of each marker in each cell was determined based on the bimodal distribution of fluorescence intensity among all segmented cells. Different cell types were defined as follows: CD8+ T cell (CD8+ CD45+), tumor‐associated macrophage (F4/80+ CD45+), dendritic cell (CD11c+ CD45+), pan‐hematopoietic cell (CD45+). Cell Profiler (Ver 4.1.3), FIJI, Prism 9, and Python (Ver 3.7.0) were used for cell type annotation and data presentation.

### Statistics

GraphPad Prism was used for statistical analysis. Results were expressed as mean ± SEM. Statistical tests included one‐way ANOVA followed by Tukey's or Dunnett's multiple comparison test. When applicable, the unpaired one‐tailed and two‐tailed Student's t‐tests using Welch's correction for unequal variances were used.

## Conflict of Interest

RW is a consultant to ModeRNA, Lumicell, Seer Biosciences, Earli, and Accure Health, none of whom contributed to this research. The other authors report no affiliations.

## Author Contributions

R.D. and E.A.H. contributed equally to this work. R.W. performed conceptualization. R.D., E.A.H., J.O., R.H.K., and I.R.F. performed data acquisition. All authors performed data analysis. E.A.H. and R.D. performed synthesis. E.A.H. and R.W. performed supervision. R.W. performed visualization. R.W. wrote original draft. R.W. and all coauthors wrote, reviewed and edited. R.W. performed funding acquisition, project administration andacquired resources.

## Supporting information

Supporting Information

Supplemental Movie 1

## Data Availability

The data that support the findings of this study are available from the corresponding author upon reasonable request.
