# Supplementary material for: Hybrid LNP Prime Dendritic Cells for Nucleotide Delivery
Source: Adv Sci (Weinh). 2023 Oct 9;10(33):2303576. doi: 10.1002/advs.202303576 (PMC10667837; doi:10.1002/advs.202303576)
Supplement: Supplementary file 1 — Supporting Information [file ADVS-10-2303576-s002.pdf]

ADVANCED SCIENCE
Open Access

Supporting Information

for *Adv. Sci.*, DOI 10.1002/advs.202303576

Hybrid LNP Prime Dendritic Cells for Nucleotide Delivery

*Riddha Das, Elias A. Halabi, Ina R. Fredrich, Juhyun Oh, Hannah M. Peterson, Xinying Ge, Ella Scott, Rainer H. Kohler, Christopher S. Garris and Ralph Weissleder**

Supplemental information

Hybrid LNP prime dendritic cells for nucleotide delivery

Riddha Das[1]*, Elias A. Halabi[1]*, Ina R. Fredrich[1], Juhyun Oh[1], Hannah M. Peterson[1], Xinying Ge[1], Ella Scott[1], Rainer Kohler[1], Christopher S. Garris[1,2], Ralph Weissleder[1,3,4#]

[1] Center for Systems Biology, Massachusetts General Hospital, 185 Cambridge St, CPZN 5206, Boston, MA 02114,

[2] Department of Pathology, Massachusetts General Hospital, Boston, MA 02114

[3] Department of Radiology, Massachusetts General Hospital, Boston, MA 02114

[4] Department of Systems Biology, Harvard Medical School, 200 Longwood Ave, Boston, MA 02115

* Equal contributions

# corresponding author:

*R. Weissleder, MD, PhD
Center for Systems Biology
Massachusetts General Hospital
185 Cambridge St, CPZN 5206
Boston, MA, 02114
617-726-8226
rweissleder@mgh.harvard.edu

Fig. S1: L-CANDI Characterization. A. Three-step synthesis to loaded L-CANDI particles starting from 1) cross-linking of succinyl-beta-cyclodextrin (s-**β**-CD) and L-Lysine with EDC/NHS, 2) surface functionalization of nucleophilic groups with palmitic acid NHS ester in carbonate buffer and 3) payload (LCL-161) adsorption on particle's surface via inclusion-complexation with CD units. **B.** Plots showing the size distribution and surface charge of all particles obtained from dynamic light scattering (DLS) and zeta measurements, respectively (Zetaziser, Malvern). All experiments were performed in triplicates (N = 3).

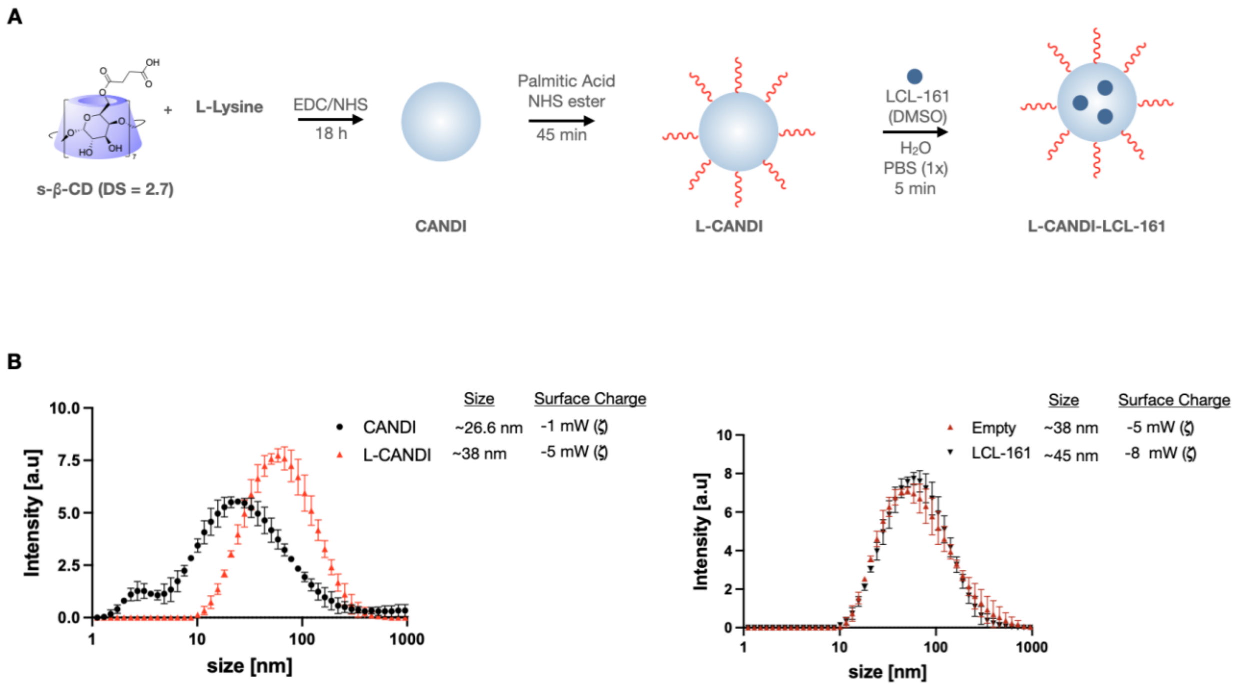

Fig. S2: C12-200 Characterization. A. ^{1}H-NMR of C12-200 in $CDCl_3$ showing the structure, chemical formula, and exact mass (ChemDraw 20, Perkin Elmer). **B**. HPL chromatograph with (ELDS source) of the purified product shows a single peak with a retention time of t = 3.65 min and ionizes with an m/z of ES^+ = 1137.24 {M^{+}}.

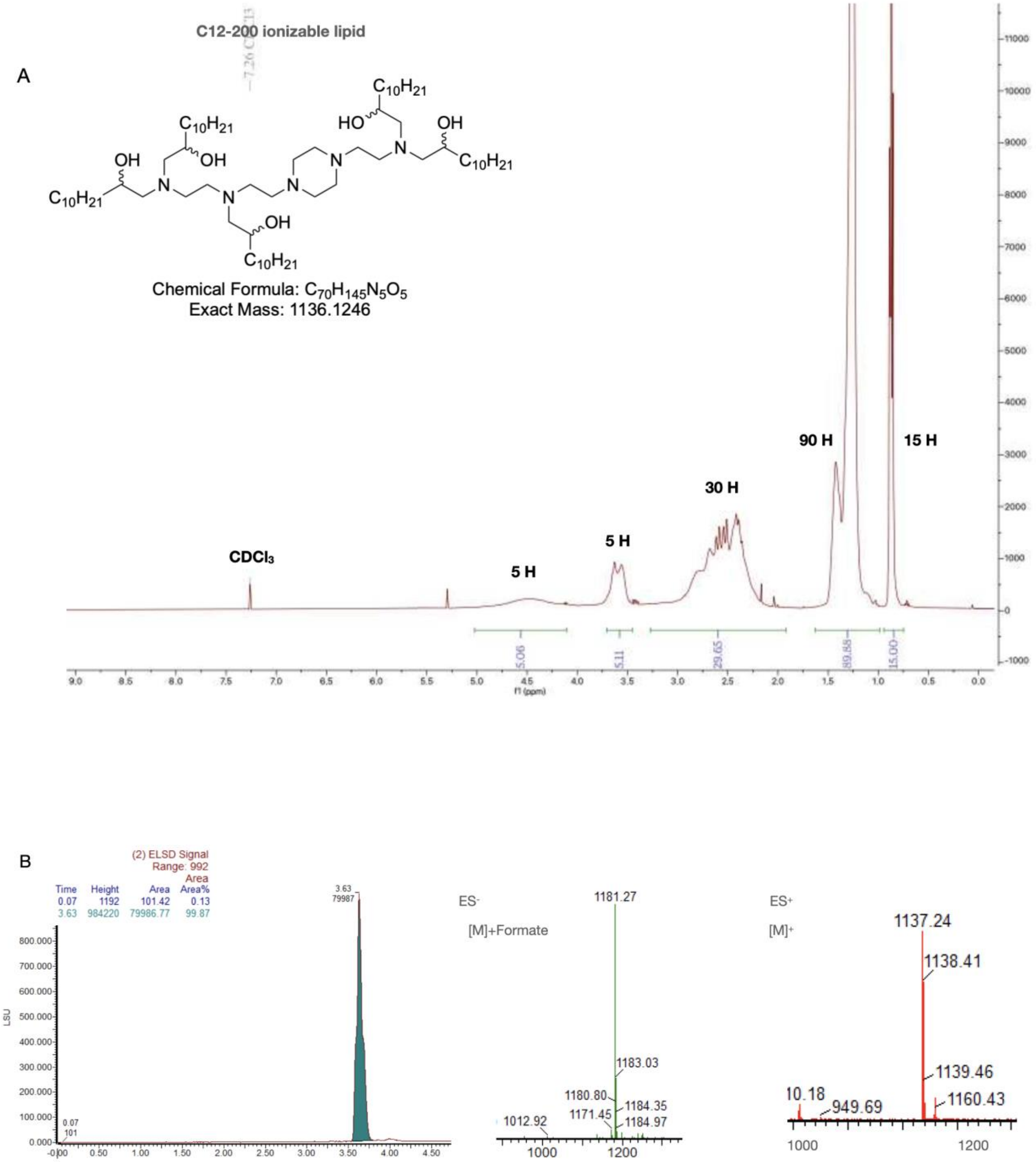

Fig. S3: Direct LCL-161 incorporation into LNPs leads to rapid release. A. Full liquid chromatograph of LNP solution encapsulating LCL-161, right after mixing (t = 0 min) and after 4 washing steps (10 min each). All washing step were performed using an amicon centrifugation filter (50 KDa, 10 min, 2000 rpm). Right plot shows a zoomed in area of the peak corresponding

to LCL-161 (retention time 0.94 min), total injected volume (10 µL). The area under the curve obtained after each washing step was used to **B**. calculate the percentage of retained LCL-161 after each step normalized to the initial detected amount "pre-wash" and **C**. determine the total amount in mg of retained LCL-161. **D**. Pictures of vials containing a typical colloidal LNP solutions before and after washing to showcase that the washing procedure does not dilute or break the nanoparticles. All experiments were performed in triplicates (n = 3).

Fig. S4: Inclusion-complexation assay determines L-CANDI incorporation in LNPs. A. Schematic depiction of the assay used to quantify the total amount of encapsulated L-CANDI in LNP15 (without poly I:C). LNP1 (1 mg/mL) was used as a negative control and compared to LNP15 (1 mg/mL) containing L-CANDI without poly I:C. Bisbenzimide H 33258 (Hoechst) was

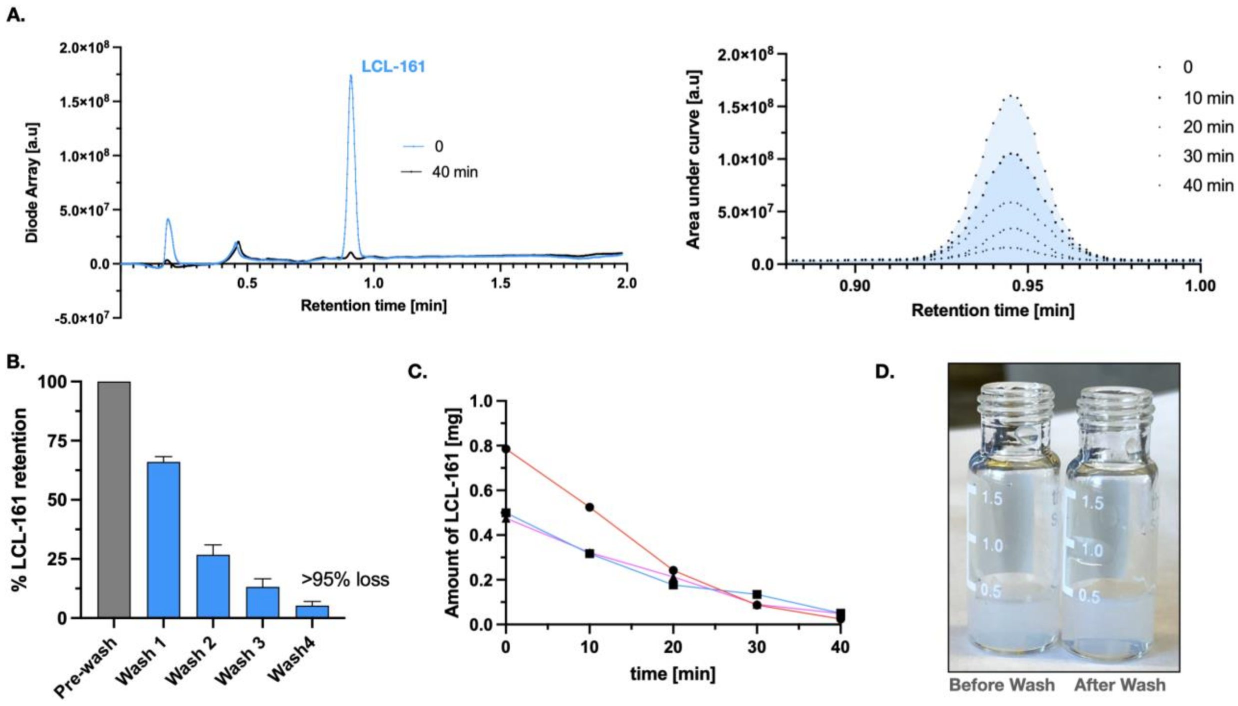

used as a membrane-permeable fluorescent indicator selective for the CD units in L-CANDI resulting in increased fluorescence emission at 510 nm. **B**. Standard deviation curve of a fixed Hoechst concentration (0.05 ng/mL) with varying L-CANDI concentration (100–3200 µg in PBS, 1x). **C**. Graph of the analyzed solutions containing LNP1 and LNP15 (100 µL). The encapsulation efficiency of L-CANDI in LNP15 was 18.6±2.2%. All experiments were performed

in triplicates (N = 3) and compared using unpaired student's t-test (***, p = 0.0001) subtracting the background fluorescence from the PBS control.

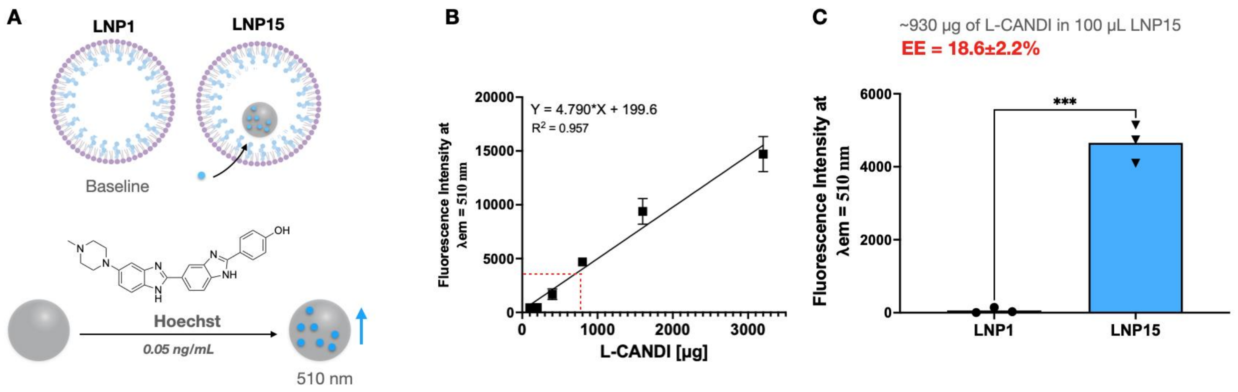

Fig. S5: Stability of LNP stored at 4°C **A.** Size as a function of time. **B**. Polydispersity was measured by DLS **C**. Poly I:C encapsulation efficiency was determined by the RiboGreen assay. **D.** stability of LNP17 in the presence of 0, 10, 25% fetal bovine serum measured by DLS. Note that LNP17 exhibited long-term stability with no evidence of aggregation in the presence of serum.

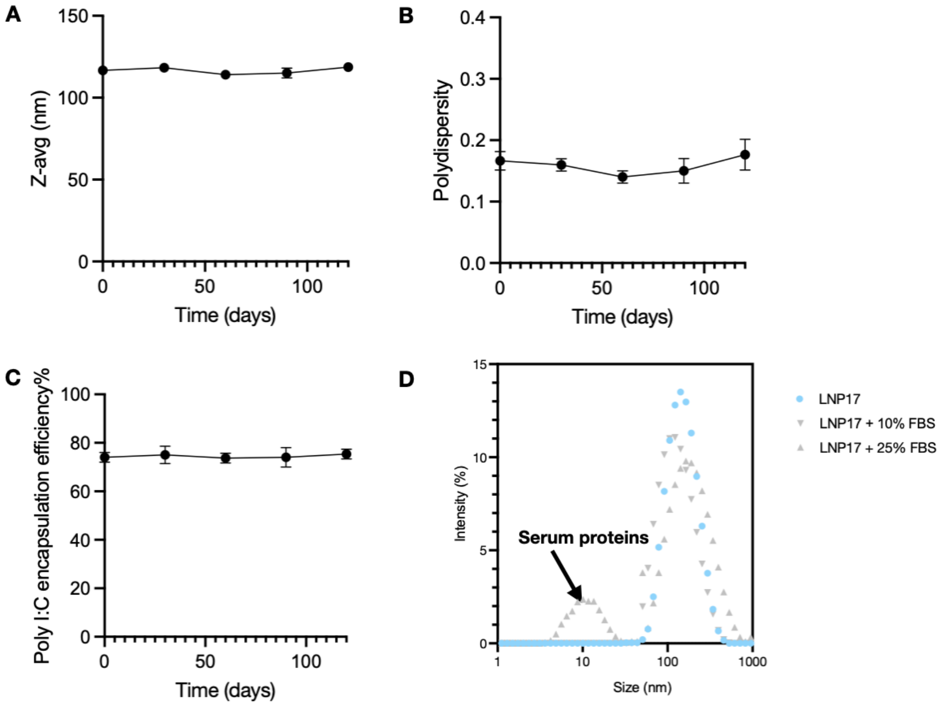

Fig. S6: Comparison of efficacy (IL-12 induction in BMDM) and cellular toxicity (iMAC). A. **A.** IL-12 induction measured in BMDM. **B.** Cellular toxicity in iMAC as a function of dose. The

shaded area in green represents the therapeutic window. The in vivo dose (5 µg poly I:C/mouse in 0.774 mL of plasma corresponds to a vascular concentration of 6.5 µg/mL(1)) is denoted by the red dotted line. There is cellular toxicity at very high doses (> 100x used therapeutically) due to the combination of LCL-161 and poly:IC. The LNP preparation is not toxic at these doses.

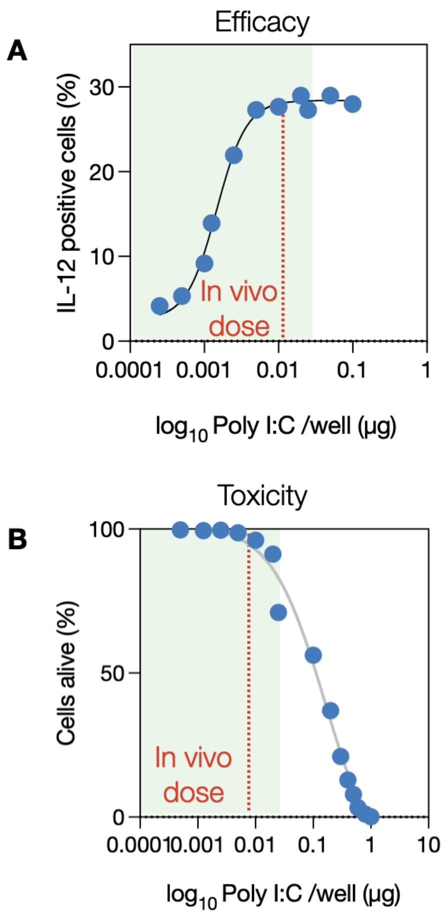

Fig. S7: Blood half-life of LNP. **A.** LNP17Fl or PBS control was injected into BL6 mice and serial imaging of the ear vasculature was performed. Blood half-life (T1/2) was determined to be 30 min. B. Time-series of images depicts the fluorescent LNP17Fl in the ear microvasculature.

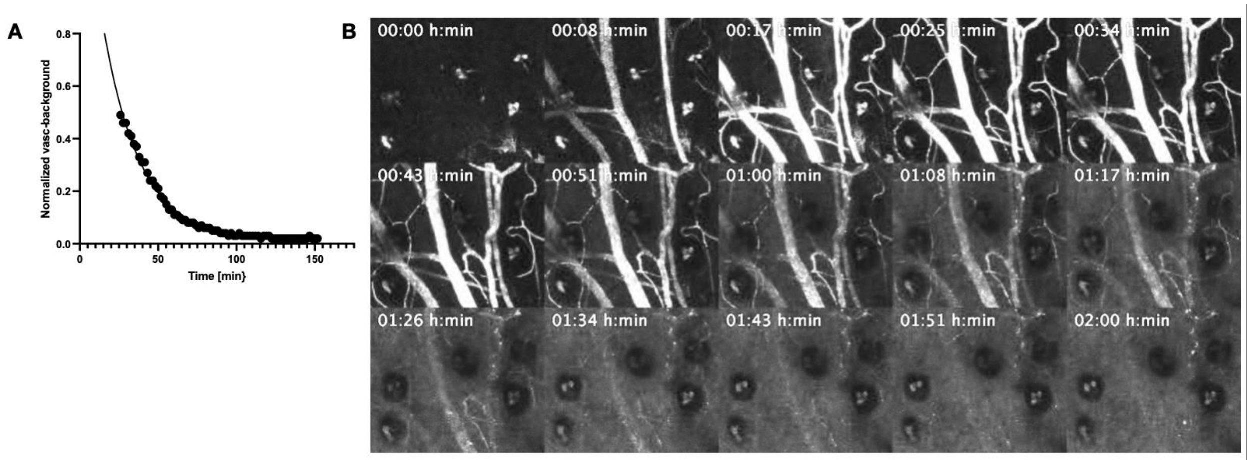

Fig. S8: Tissue distribution of LNP. **A.** LNP17Fl or PBS control was injected into tumor-bearing mice (MC38; N = 6 mice), and tissues were surgically removed 24 hr later. The graph shows the relative distribution of LNP to different organs. As expected, liver and spleen tumor lymph nodes showed the highest concentrations. **B**. Tissues were imaged using an OV100 epifluorescence system. Bright signal (647 nm) corresponds to fluorescent LNP17 accumulation. **C**. Flow cytometry data. LNP17Fl was injected into tumor-bearing mice (MC38; N = 6 mice), and tissues were surgically removed 24 hr later. The graph shows the relative distribution of LNP to different organ cells as measured by flow cytometry, confirming the fluorescence imaging experiments.

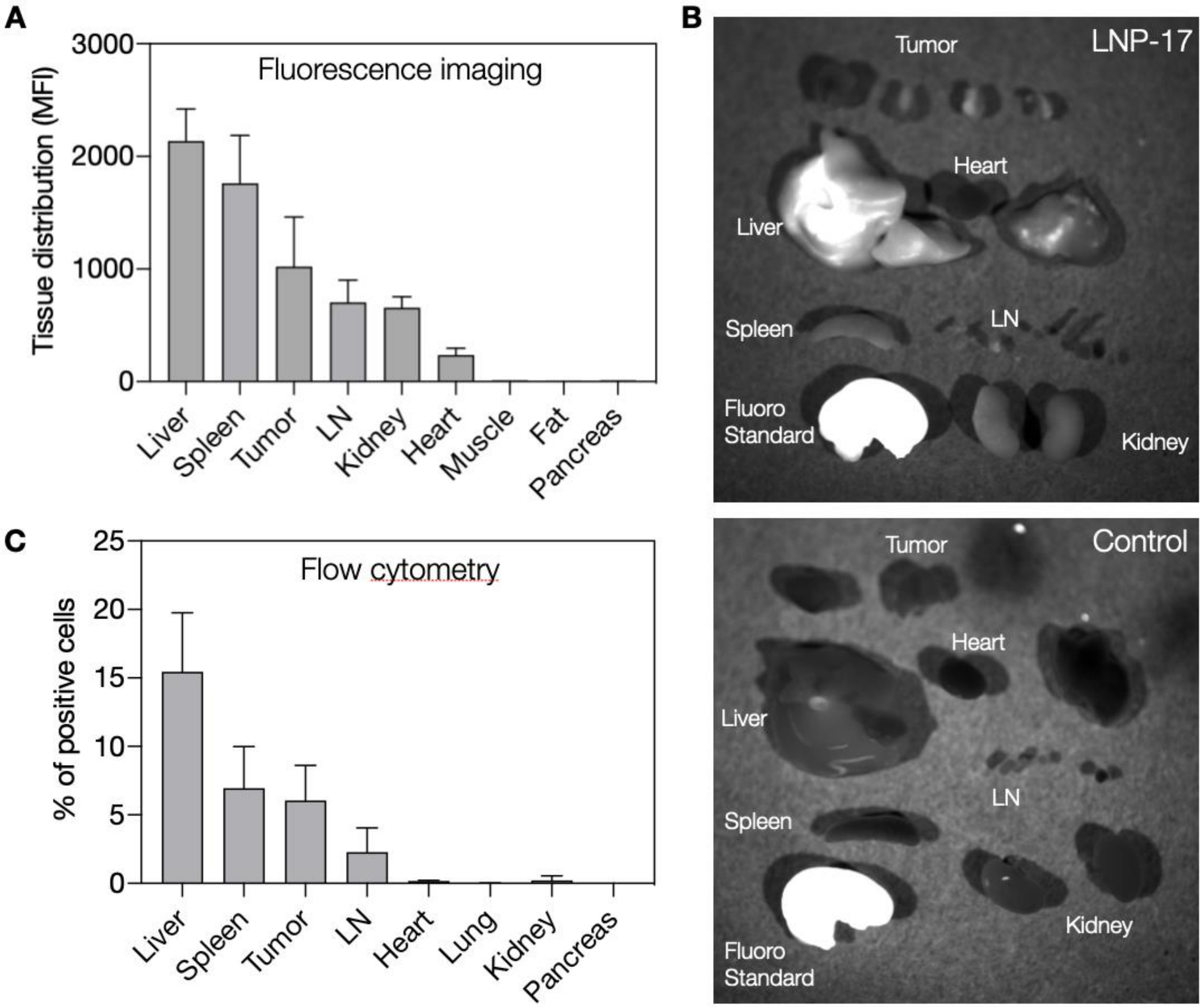
A
Fluorescence imaging
Tissue distribution (MFI)
3000
2000
1000
0
Liver
Spleen
Tumor
LN
Kidney
Heart
Muscle
Fat
Pancreas
B
LNP-17
Tumor
Heart
Liver
Spleen
LN
Fluoro Standard
Kidney
Control
Tumor
Heart
Liver
LN
Spleen
Kidney
Fluoro Standard
C
Flow cytometry
% of positive cells
25
20
15
10
5
0
Liver
Spleen
Tumor
LN
Heart
Lung
Kidney
Pancreas

Fig. S9: Tumoral accumulation. **A.** LNP17Fl was injected into tumor-bearing mice (MC38; N = 3 mice), and tumors were imaged serially over 24 hr. The LNP is initially seen in tumor microvasculature but accumulates in non-tumor cells at 24 hr. Scale bar = 100 µm.
Fig. S10: Dynamic changes in the tumor immune microenvironment induced by fluorescent LNP17 . A. Representative images of MC38 tumors with a fluorescent LNP17Fl

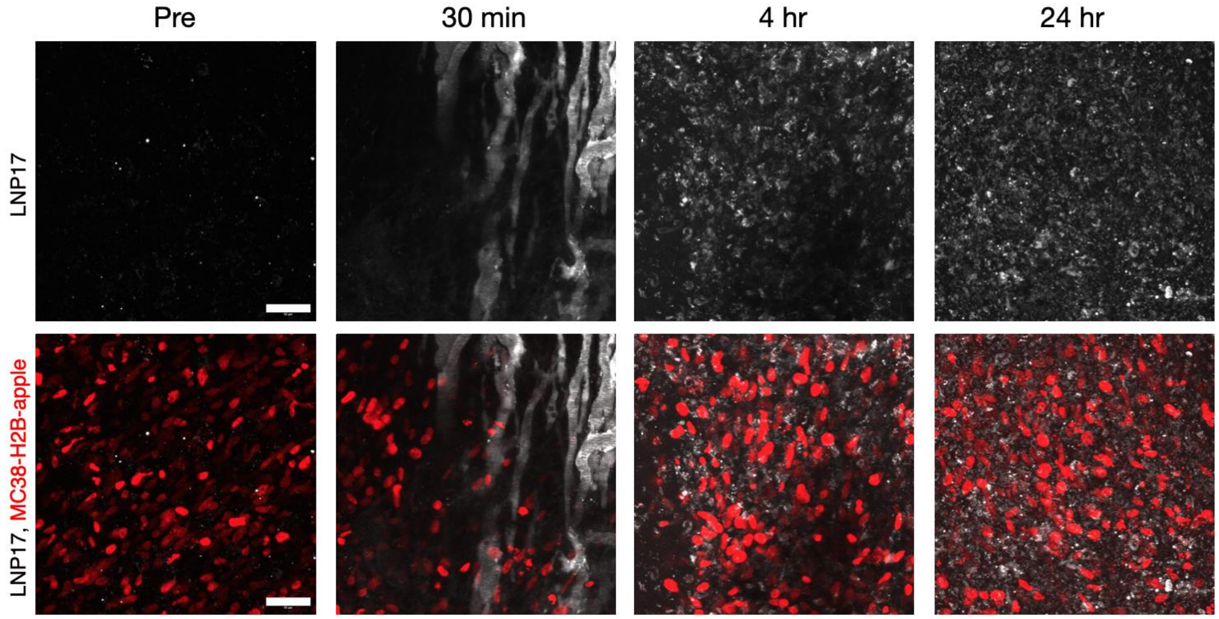

(LNP17 with ATTO647N) treatment (top row) or without treatment (bottom row) show increased infiltration of CD8+ T cells (red) and dendritic cell (DC, yellow) populations in tumors. F4/80+ tumor-associated macrophage (TAM) is shown in cyan, and fluorescent LNP17 is in grey. Scale bar = 100 µm. **B.** Quantification of immune cell infiltration in tumors with or without LNP17Fl treatment. The frequency of F4/80+ TAM, CD11c+ DC, and CD8+ T cells among CD45+ cells and the frequency of CD45+ cells among all cells with DAPI+ nucleus were measured for tumors from LNP17Fl-treated and control mice.

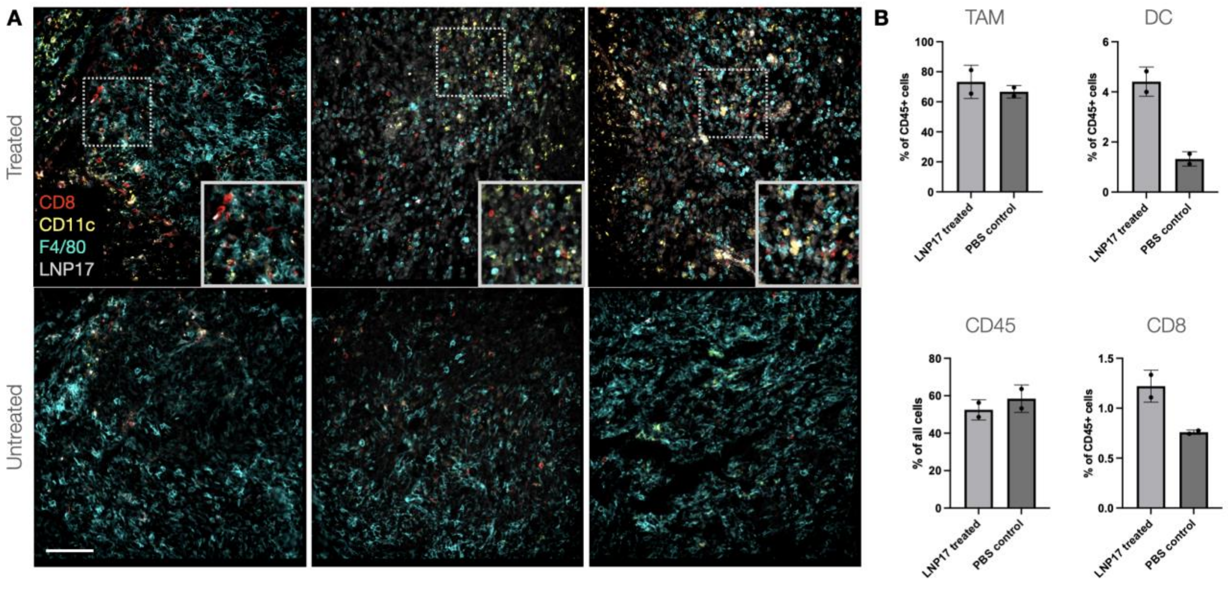

Fig. S11: Spatial analysis of immune cell subsets in LNP17 treated (A) or untreated (C) tumors. For each specimen, 12 representative fields of views are shown. Gray marks, CD45+ cells and blue dots represent superimposed, dendritic cells. Note the higher DC infiltration in

LNP17 treated tumors. **B** Quantitation of DC in LNP17, treated and control tumors. **D.** Quantitation of CD8 in LNP17, treated and control tumors.

MOVIE

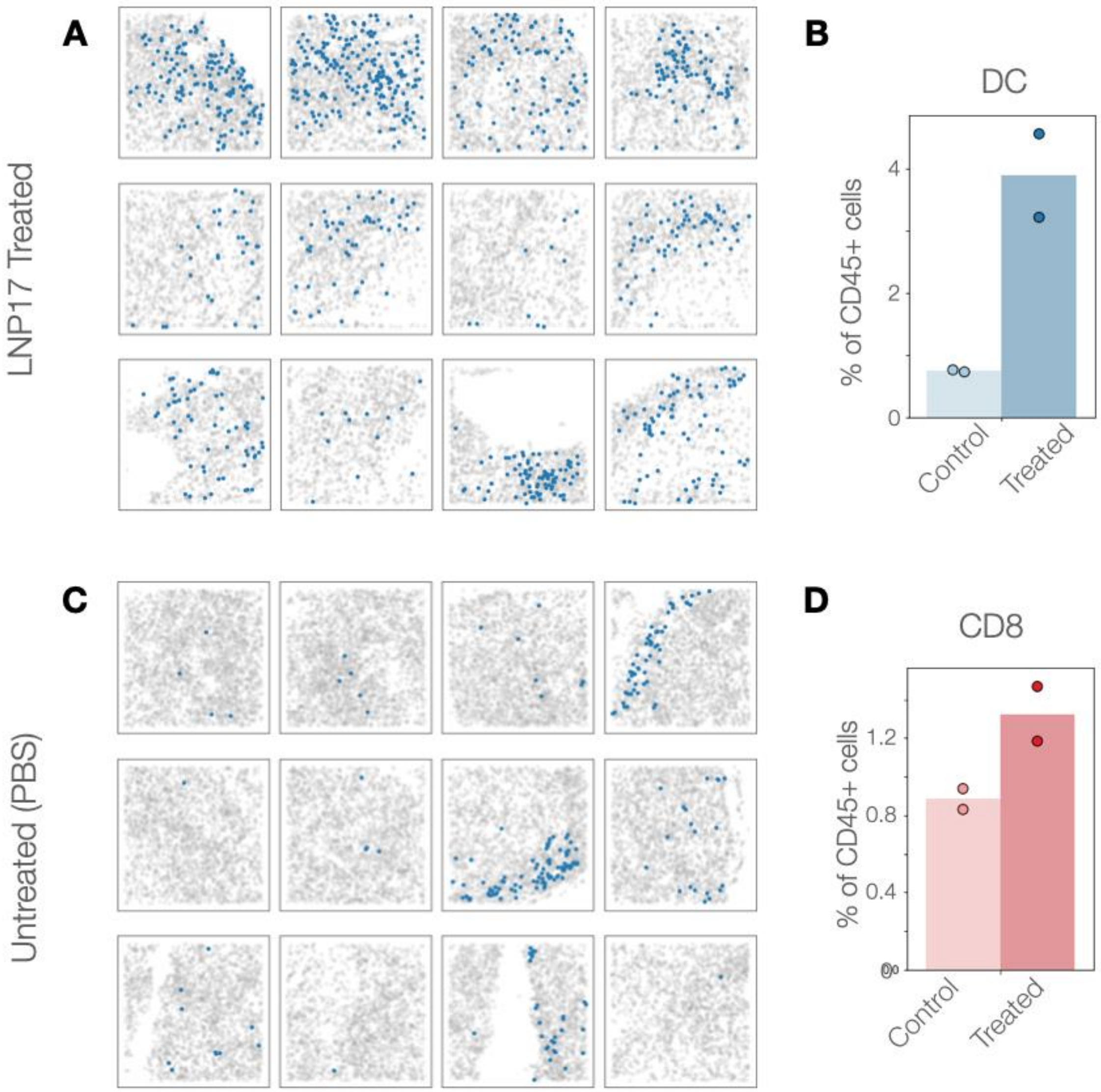

Movie S1: LNP17Fl or FITC-dextran control was IV injected into BL6 mice and serial imaging of the ear vasculature was performed.

TABLES

**Table S1: Overview of hybrid LNP tested (only preparation in bold were tested in cells and/or in vivo). ***4 lipid composition: C12-200, cholesterol, DSPC, mPEG-DMG in a 35:16:46.5:2.5 ratio. PGA: polyglutamic acid. Poly I:C: 0.2-1 kb

LNP ID	Composition*	Condenser	Nucleic acid	Hybrid NP	Stain
**LNP1**	4 lipids: *	None	None	None	None
LNP2	4 lipids: *	PGA	None	None	AF555 to stain lipid
LNP3	4 lipids: *	PGA	None	None	AF647 to stain lipid
LNP4	4 lipids: *	PGA	None	None	AF555 to stain lipid
LNP5	4 lipids: *	PGA	None	None	AF647 to stain lipid
LNP6	4 lipids: *	PGA	None	None	Cy5 to stain lipid
LNP7	4 lipids: *	PGA	None	None	Rhodamine to stain lipid
LNP8	4 lipids: *	PGA	None	None	Cy5 to stain lipid
LNP9	4 lipids: *	None	None	None	Cy5 to stain lipid
LNP10	4 lipids: *	None	None	None	DiL to stain lipid
LNP11	4 lipids: *	PGA	None	None	Atto647N to stain lipid
LNP12	4 lipids: *	PGA	None	None	Atto647N to stain lipid
LNP13	4 lipids: *	None	None	None	Atto647N to stain lipid
**LNP14**	4 lipids: *	Poly I:C	Poly I:C	None	None
**LNP15**	4 lipids: *	None	None	L-CANDI-117	None
**LNP16**	4 lipids: *	Poly I:C	Poly I:C	L-CANDI-115	None
**LNP17**	4 lipids: *	Poly I:C	Poly I:C	L-CANDI-117	None
**LNP17Fl**	4 lipids: *	Poly I:C	Poly I:C	L-CANDI-117	Atto647N for lipid
**LNP17EM**	4 lipids: *	Poly I:C	Poly I:C	L-CANDI-117	Uranyl acetate of L-CANDI for EM

Table S2: Antibodies used in flow cytometry analysis

Markers	Target	Clone	Source	Catalogue#	Dye
CD45	Leukocytes	30-F11	Invitrogen	56-0451-82	AF700

Markers	Target	Clone	Source	Catalogue#	Dye
MHCII	APC	M5/114.15.2	Invitrogen	47-5321-82	APC-eFlour 780
Tim4	Kupffer cells	RMT4-54	BioLegend	130005	PE
CD11c	DC	N418	BioLegend	61-0114-82	PE-eFlour 610
F4/80	TAM	BM8	Invitrogen	25-4801-82	PE-Cy7
CD64	Neutrophils	X54-5/7.1	BioLegend	139309	BV421
CD11b	Myeloid cells	M1/70	BioLegend	101239	BV650
CX3CR1	Myeloid cells	SA011F11	BioLegend	149029	BV785
CD19	B cells	e-Bio 1D3	BioLegend	15-0193-81	PE-Cy5
B220	B cells	RA3-682	Invitrogen	15-0452-81	PE-Cy5
CD11b	Myeloid cells	M1/70	BD Biosciences	557657	APC-Cy7
Ly6C	Monocytes	HK1.4	BioLegend	128031	BV421
Ly6G	Neutrophils	1A8	BioLegend	127643	BV711
CD3e	T cells	145-2C11	BioLegend	100320	PE-Cy7

Table S3: Antibodies Used for Histology

Markers	Target population	Clone	Source	Catalog #	Dye
CD45	Pan hematopoietic cells	104.2	BioXcell	BE0300	AF488
CD8	CD8+ T cells	53-6.7	Biolegend	100702	AF555
CD4	CD4+ T cells	RM4-5	Biolegend	100505	AF488
F4/80	Macrophages	CI:A3-1	BioXcell	BE0206	AF555
CD11c	Dendritic cells	N418	Biolegend	117302	AF488
CD31	Various	MEC13.3	Biolegend	102520	AF594

References:

1. Zhu, H., Melder, R. J., Baxter, L. T., and Jain, R. K., Physiologically based kinetic model of effector cell biodistribution in mammals: implications for adoptive immunotherapy., *Cancer Res*, *56*, 3771-3781 (1996).
